# Optical coherence tomography angiography for the detection and evaluation of ptic disc neovascularization: a retrospective, observational study

**DOI:** 10.1186/s12886-022-02351-9

**Published:** 2022-03-16

**Authors:** Xiang-ning Wang, Jun Zhou, Xuan Cai, Tingting Li, Da Long, Qiang Wu

**Affiliations:** 1 Department of Ophthalmology, Shanghai Jiao Tong University Affiliated Sixth People’s Hospital, 600 Yishan Road, Shanghai, 200233 China; 2 Shanghai Key Laboratory of Diabetes Mellitus, Shanghai, 200233 China

**Keywords:** Optic disc neovascularization, Optical coherence tomography angiography, Vitreoretinal interface, Proliferative diabetic retinopathy, Imaging

## Abstract

**Background:**

To assess and characterize neovascularization of the optic disc (NVD) using optical coherence tomography angiography (OCTA) and different OCTA-based methods.

**Methods:**

This retrospective, observational study included patients who were suspected of having early PDR with no presence of clinically apparent neovascularization (NV) bur were clinically diagnosed with proliferative diabetic retinopathy (PDR), or severe NPDR. Patients underwent standard clinical examinations and OCTA imaging using a 6 × 6 montage scan. Two trained graders identified NVD using different imaging systems (ultra-widefield-colour fundus photography (UWF-CFP), OCT, OCTA and fluorescein angiography (FA)). Moreover, morphological classification of NVD was performed. The detection and morphological classification of NVD by different OCTA-based methods (B-scan OCTA, En-face OCTA, VRI Angio and VRI Structure) were compared.

**Results:**

A total of 169 eyes (126 eyes with PDR and 43 eyes with severe NPDR) of 123 participants were included in this study. The detection rate of NVD was 34.91% by UWF-CFP compared with 59.76% by OCT, 59.76% by OCTA, and 62.72% by FA. After excluding 2 cases with epiretinal membranes, the NVD diagnosis detected by OCT was used as the standard. Among 99 eyes diagnosed with NVD by OCT, B-scan OCTA detected NVD with a sensitivity of 97.98%, which was higher than that by en face OCTA (80.81%), VRI Angio (65.66%), and VRI Structure (61.62%) (all *P* < 0.05). According to its characteristics on OCTA, NVD was divided into four types (12 cases of type I, 6 cases of type II, 39 cases of type III, and 42 cases of type IV). For type I, B-scan OCTA exhibited a higher diagnostic sensitivity than other methods (*P* < 0.05). For types II and IV, there were no statistically significant differences in the sensitivity of various methods between the two groups (*P* > 0.05).

**Conclusion:**

OCTA and different OCTA-based methods are significant to the diagnosis of NVD, and the diagnostic accuracy of different detection methods may be related to different types of NVD.

## Background

Neovascularization of the optic disc (NVD) refers to the formation of new blood vessels in the optic disc, which frequently occurs among patients with proliferative diabetic retinopathy (PDR). It is one of the main causes of severe vision loss. According to previously reported findings, patients with PDR and NVD are at a significant risk of vision loss. NVD accounts for 85% of PDR haemorrhages. Half of PDR patients with untreated NVD become blind within 3 years after the initial diagnosis [1]. As a result, early identification and timely treatment of NVD are essential to decrease the risk of visual loss in PDR patients with NVD [2].

Diagnosing PDR is sometimes a major challenge. To our knowledge, slit-lamp fundus biomicroscopy has a low sensitivity for differentiating neovascularization (NV) from intraretinal microvascular abnormalities (IRMAs) [3]. Smaller lesions may not be detected by an examiner, especially those that induce mild PDR symptoms. Another difficulty is to conduct a comprehensive follow-up. After panretinal photocoagulation (PRP) or intravitreal injection therapy, clinical examinations may reveal symptoms of a persistent neovascular complex. In addition, tractional retinal detachment (TRD) and vitreous haemorrhage (VH) present challenges related to surgical decision-making [4].

Colour fundus photography (CFP) and fluorescein angiography (FA) were previously used to assess patients at different stages of diabetic retinopathy (DR) and to further plan patient treatment. Ultra-widefield (UWF)-CFP is associated with some deficiencies, such as the inability to differentiate pseudo-colours, peripheral distortion, and reduced resolution. Additionally, CFP cannot distinguish nonperfusion areas (NPAs) from NV areas, and differentiating IRMAs from NV is challenging [5–8]. FA is currently the gold standard for the diagnosis and staging of DR lesions, because it can accurately identify vascular lesions, such as micro-aneurysms (MAs), the extent of regions of nonperfusion and the foveal avascular zone (FAZ), and retinal NV [9]. Although FA has a well-known diagnostic capability, it is an invasive, time-consuming, and expensive treatment that may cause infrequent and severe dye-related side effects. These limitations are especially important for diabetic patients who have impaired renal function or are pregnant, as they are at a higher risk of developing DR. Furthermore, since FA is restricted to two-dimensional information, it may be unable to distinguish whether leakage is caused by NV and blood vessels breaching the internal-limiting membrane (ILM) into the vitreous, which is a characteristic of PDR, or by IRMAs inside the retina, which is a sign of non-PDR (NPDR) [10].

Optical coherence tomography angiography (OCTA) has facilitated non-invasive imaging of retinal and choroidal microvasculature abnormalities associated with DR, including MAs [11], NPAs [12], IRMAs [13], NV [14] and choriocapillaris flow deficits [15]. The recently developed widefield swept-source (WF-SS)-OCTA system has remarkably extended the field of view (FOV) to 50–80° of the retinal surface, which has the potential to change the existing DR diagnosis and follow-up based on the CFP and FA paradigms.

Several recent studies have proven the effectiveness of identifying NV via SS-OCTA vitreoretinal interface (VRI) slab images, which capture extraretinal lesions extending into the vitreous [16, 17]. Lu et al. compared the sensitivity of the WF-SS-OCTA-VRI Angio slab and the SS-OCT-VRI Structure slab in detecting diabetic retinal NV. They discovered that the VRI slab might be beneficial for diagnosing and monitoring PDR in clinical practice [18].

To date, numerous studies have concentrated on the categorization of NVD based on OCTA. Khalid H et al. [19] classified NVD into four types based on OCTA. An OCTA B-mode scan for type 1 NVD reveals a preretinal hyperreflective material (PRHM) that connects the physiological cup to a visible flow signal on the superficial slab, allowing the signal to engulf the physiological cup. In the B-mode scan, Type 2 NVD appears as a tiny bud, which may be visible on the VRI slab. In type 3 NVD cases, the PRHM may be visible on the surface, and may also be identified on the VRI slab. As seen in the B-mode scan, the PRHM in type 4 NVD is raised above the surface.

## Methods

### Data collection

This prospective, observational study was conducted in the Department of Ophthalmology at Shanghai Sixth People’s Hospital from January 2010 to June 2021. Patients who were suspected of having early PDR with no presence of clinically apparent NV but were clinically diagnosed with PDR (either naïve or previously treated) or severe NPDR. Exclusion criteria included eyes with concomitant chorioretinal diseases, glaucoma and severe media opacities, which interfered with image acquisition; poor image quality that prevented the device from automatically creating montage images; or image quality that was otherwise determined to be insufficient for clinical assessment (e.g., due to poor fixation or image artefacts).

The study was approved by the Institutional Review Board of Shanghai Sixth People’s Hospital. All protocols were in accordance with the principles of the Helsinki Declaration, and written informed consent was obtained from the patients prior to their participation in the study.

### Study protocol

All included participants underwent ophthalmic examinations, which included obtaining a medical history (gender, age, history of diabetes, blood glucose, history of previous treatment, etc.) determining visual acuity, slit lamp biomicroscopy of the anterior segment, ophthalmoscopy of the posterior segment, and wide-field colour fundus imaging using the Optos camera (Optos PLC, Dunfermline, UK), FA system (Spectralis HRA + OCT; Heidelberg Engineering GmbH, Heidelberg, Germany), and OCT/OCTA system (Heidelberg Engineering GmbH). UWF-CFP, FA, and OCT/OCTA were performed on the same day or within 1 week.

### Image acquisition protocol

OCTA scans were conducted out using the OCT/OCTA system (Heidelberg Engineering GmbH). A laser with a central wavelength of approximately 870 nm was used in the OCT/OCTA system (Heidelberg Engineering GmbH) at a scan rate of 85,000 A-scans/s.

Regarding the scanning protocol in our clinic, for each patient, a high-definition 6-mm line scan and a 6*6-mm volume scan that were centred on the optic nerve head were acquired (FOV of 20*20°). Each 6*6 mm volume had 512 A-scans per B-scan (with a space of 24 mm between adjacent A-scans) and 512 B-scan positions per volume scan (with a space of 11 mm between adjacent B-scans); OCTA images were acquired using two replicated B-scans at each B-scan spot. It took at least 12 s to obtain a single 6*6 mm volume. Professional ophthalmic photographers performed all OCTA scans, and in necessary cases, they replicated the acquired images several times to ensure that high-quality photographs of high-intensity OCT signals and minimum motion artefacts were captured. The quality scores of scans were expressed as a signal-to-noise ratio (SNR) in decibels (dB) on a scale of 1 (poor quality) to 40 (excellent quality), and the included scans had a score > 20 dB, which was considered good quality. The FA photographs were acquired using the Heidelberg Spectralis HRA + OCT system (Heidelberg Engineering GmbH).

NVD was detected using the following methods: (1) biomicroscopy or CFP showed the presence of clearly formed NVD; (2) structural OCT displayed the presence of PRHM; and (3) with the presence of PRHM on structural OCT, an OCTA scan was conducted in that location. A diagnosis of NV was made if a flow signal was evident on the B-scan OCTA or a clear NVE could be observed on the en face OCTA; (4) the presence of leakage on FA was recorded.

**Fig. 1:**
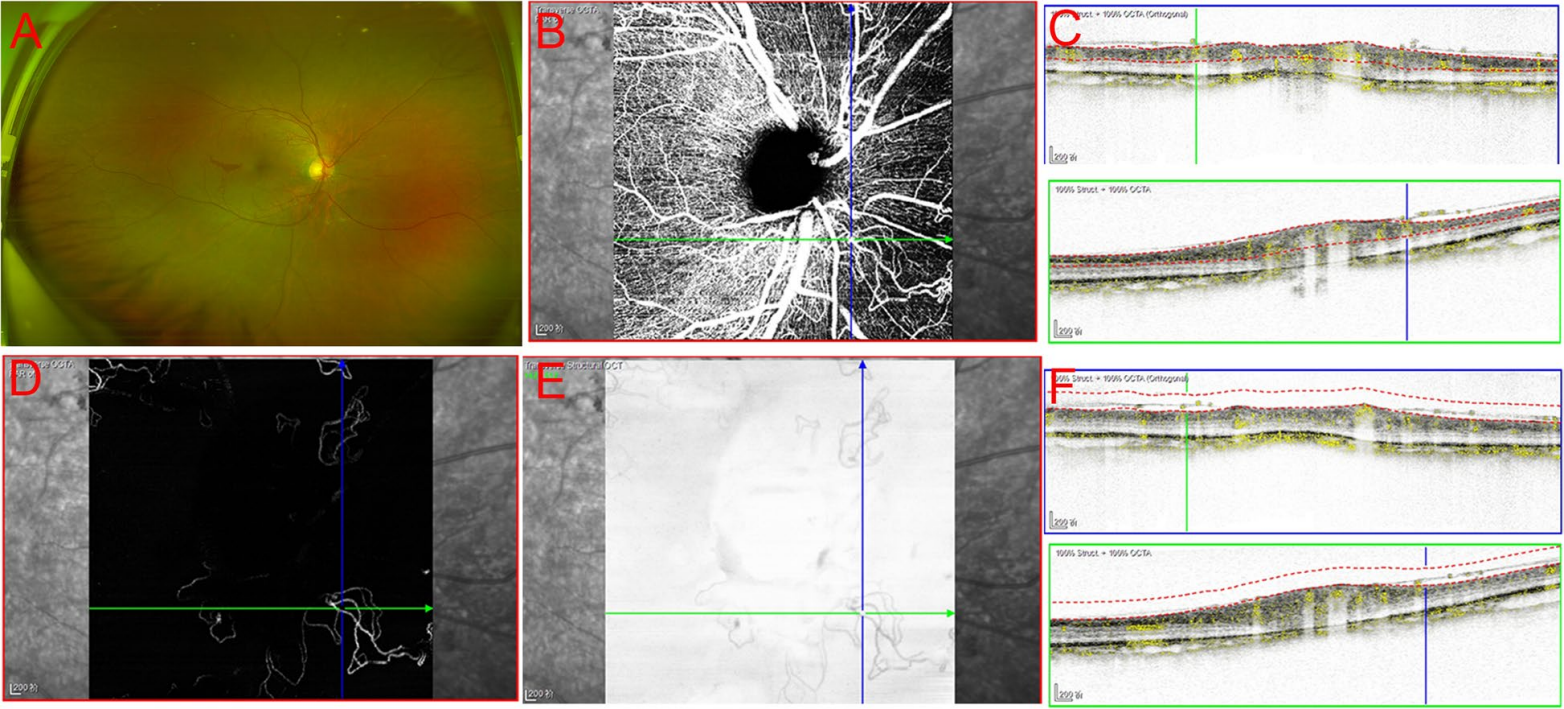
Detection of neovascularization of the disc (NVD) in patients with proliferative diabetic retinopathy (PDR). **A**, CFP showed the presence of a clearly formed NVD. **B**, en face OCTA images of the superficial retinal slab and vitreoretinal slab showed a retinal neovascular network. **C**, B-scan with PRHM detected internal blood flow signal. **D** and **E**, The VRI structure (**E**) and VRI angio (**D**) indicated that new blood vessels had developed. **F**, The VRI slab was defined with an inner boundary 100 mm above the ILM and an outer boundary at the ILM

Two qualified ophthalmologists (CX and LTT) independently examined the OCT, OCTA, and FA images for the presence of NVD, as shown on each imaging mode. Thorough examination of individual OCT B-scans within the volume for the OCT posterior pole revealed the existence of any PRHM. The built-in software of OCTA was employed to distinguish each B-scan mode in the 6*6 mm scanning area. First, a B-scan with PRHM detected an internal blood flow signal, or a clear NVE could be observed on en face OCTA. Then, the en face OCTA images of the superficial retinal slab and vitreoretinal slab were examined for obvious delineation of the retinal neovascular network. The VRI slab was defined with an inner boundary of 100 µm above the ILM and an outer boundary at the ILM. Then, the VRI Structure and VRI Angio were utilized to indicate whether any new blood vessels could be generated (Fig. 1). Morphological classification of NVD, which was previously described by Khalid et al., was performed [19].

### Statistical analysis

Statistical analyses were performed using version 18.0 SPSS statistical software (SPSS, Inc., Chicago, IL, USA). Normally distributed continuous variables are presented as the means ± SD. Differences in NVD detection rates among different groups were compared using the McNemar’s test or the exact McNemar’s test. For all tests, a *P* value < 0.05 was considered statistically significant.

## Results

### Patient demographic features

A total of 123 patients with 169 eyes (126 with PDR, 43 with severe NPDR) were included. It was estimated that each participant had diabetes for an average of 19.2 ± 8.5 years. The median age of the participants was 54.36 ± 17.68 years old. In addition, 82% of patients had type 2 diabetes mellitus. At the time of imaging, 105 eyes (62.13%) were untreated. Twelve eyes with PDR and 17 eyes with severe NPDR were treated with PRP, 13 eyes with PDR and 7 eyes with severe NPDR were treated with PRP and intravitreal injection, and 15 eyes with PDR were treated with vitrectomy.

### Comparing the results of OCTA

The detection rate of NVD was 34.91% by UWF-CFP compared with 59.76% by OCT, 59.76% by OCTA, and 62.72% by FA. Moreover, 5 NVE lesions were detected on UWF-FA. These lesions were not observed to perforate the ILM by B-scan and were diagnosed as leaky IRMAs.

Two epiretinal membranes were excised from 101 eyes with PRHM, which were identified via structural OCT. Among the remaining 99 eyes, 97 eyes were detected with a flow signal on the B-scan OCTA and a clear NVD on en face OCTA was found in 80 eyes. Additionally, 65 NVD eyes were identified through VRI Angio, and 61 NVD eyes were identified through VRI Structure. The diagnosis of NVD by structural OCT was used as the standard. Among the 99 eyes, B-scan OCTA detected NV with a sensitivity of 97.98%, which was higher than the detection rate by en face OCTA (80.81%), VRI Angio (65.66%), and VRI Structure (61.62%) (*P* < 0.05, Table 1). The detection sensitivity of en face OCTA was higher than that of the VRI Angio and VRI Structure (*P* < 0.05), and the difference was not statistically significant (*P* = 0.349 and 0.555, respectively).

**Table 1:** Detection rate of NVD using different OCTA-based methods

NVD	B-scan OCTA	En face OCTA	VRI angio	VRI structure	*P* value, B-scan versus En face	*P* value, B-scan versus VRI angio	*P* value, B-scan versus VRI structure
99	97.98(97/99)	80.81%(80/99)	65.66%(65/99)	61.62%(61/99)	*p* < 0.05*	*p* < 0.05*	*p* < 0.05*

*NVD* Optic disc neovascularization, *OCTA* Optical coherence tomography angiography, *VRI* Vitreoretinal interface

*The difference was of statistical significance (*p* < 0.05). McNemar test; when one or more expected values are less than 5, Exact McNemar’s test is used

Among 99 NVD eyes, there were 12 cases of type I, 6 cases of type II, 39 cases of type III, and 42 cases of type IV. In all types, a flow signal could be detected on the B-scan OCTA. For type I NVD, B-scan OCTA showed a higher detection sensitivity than other methods (P < 0.05). For type II NVD, there was no statistically significant difference in the sensitivity between the different methods (all *P* > 0.05) (Table 2). For type III NVD, B-scan OCTA exhibited a higher detection sensitivity than VRI Angio and VRI Structure (*P* < 0.05), and the difference between B-scan OCTA and en face OCTA was not statistically significant (*P* = 5.342 and 0.055, respectively). For type IV NVD, there was no significant difference in the sensitivity between methods (all *P* > 0.05) (Table 2).

**Table 2:** Different OCTA-based methods in the diagnosis of different types of NVD

Type of NVD	B-scan OCTA	En face OCTA	VRI angio	VRI structure	*P* value, B-scan versus En face	*P* value, B-scan versus VRI angio	*P* value, B-scan versus VRI structure
Type I	12	5	2	0	*p* < 0.05*	*p* < 0.05*	*p* < 0.05*
Type II	6	4	3	2	*p* = 0.455	*p* = 0.182	*p* = 0.061
Type III	39	34	22	20	*p* = 0.055	*p* < 0.05*	*p* < 0.05*
Type IV	42	37	38	40	*p* = 0.055	*p* = 0.116	*p* = 0.494

*NVD* Optic disc neovascularization, *OCTA* Optical coherence tomography angiography, *VRI* Vitreoretinal interface

^*^The difference was of statistical significance (*p* < 0.05). McNemar test; when one or more expected values are less than 5, Exact McNemar’s test is used

## Discussion

In the present study, we used OCTA to detect NVD and compared its diagnostic capability with that of UWF-CFP, OCT, and FFA. At the same time, the detection rates of different OCTA-based methods were studied, and the correlation between different types of OCTA and detection methods was also discussed.

When the diagnosis of NVD by OCT was used as the standard, UWF-CFP detected NVD with a sensitivity of 51.3%. Several factors may explain the lower detection rate of UWF-CFP [4]. First, some vessels may be extremely small and cannot be identified without high-resolution imaging. Second, bleeding may obscure NV. Finally, without the use of OCT, determining the exact position of the vessel within the retinal layers and differentiating IRMA from NV is challenging. Although UWF-CFP was suggested for complete visualization of peripheral and central retinal vascular anomalies, pseudo colours, peripheral distortion, and reduced resolution may cause erroneous detection of NVD.

By injecting fluorescent dye, dynamic images can be captured and developing changes in patients with diabetes, such as permeable vessels, can be visualized by FA, thus resulting in its recognition as the current gold standard for evaluating the retinal vasculature. However, its invasive screening and monitoring of patients with PDR hinders its popularization in clinical practice [20]. OCTA is non-invasive and may be used for all patients, even those who do not respond well to FA. OCTA is an effective technique for detecting NV in PDR patients, and it has been shown that OCTA can display the microvascular architecture of new vessels. Anti-vascular endothelial growth factor (VEGF) therapy has been extensively utilized as a safe and effective therapy for PDR [21]. SS-OCTA can be used to better characterize diabetic retinal NV alterations, measure treatment response, and make more reliable medical decisions for patients undergoing anti-VEGF therapy.

NVD may be mainly detected on B-scan OCT, followed by B-scan OCTA and en-face OCTA. NVD can be regarded as hyperreflective tissue sitting or protruding from the optic disc into the vitreous, whether the posterior hyaloid (PH) is attached or detached. It has been proven that structural OCT and B-scan OCTA provides the highest detection rate for NV. More recently, OCTA has been shown to identify the vascular structure of NVD by the presence of blood flow signals in en-face OCT angiograms [22–24].

NVD imaging requires segmentation of the retina. To identify NVD, optimized vitreoretinal segmentation procedures may cause OCTA to become an even more successful and non-invasive imaging technique [25]. Volumetric data can be segmented, and OCTA provides three-dimensional data related to the shape and geographic position of vascular lesions. Similarly, a number of studies have recently used the VRI slab imaging protocol to detect active PDR. After reconstruction of SS-OCTA-VRI slab images by manual segmentation, the sensitivity of VRI slab images for detecting NV increased to 84% [26].

**Fig. 2:**
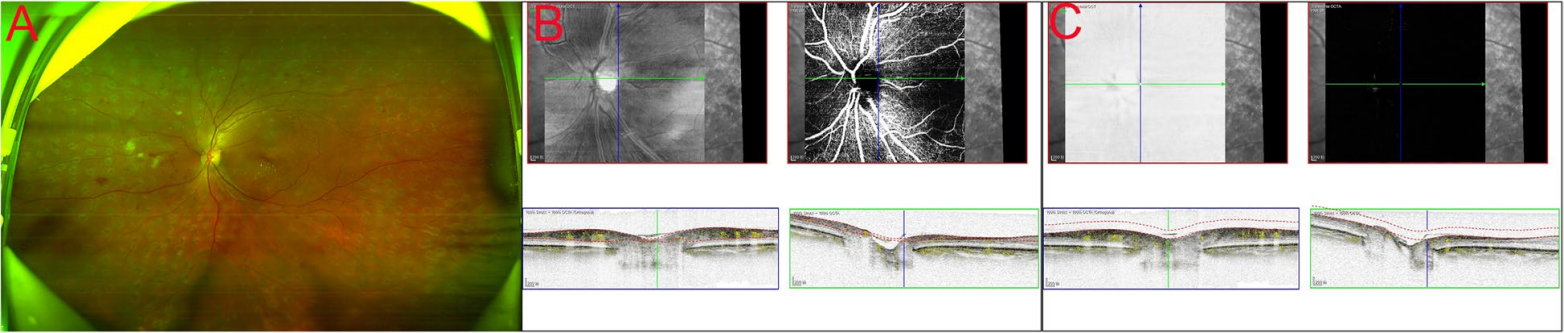
The diagnosis of type II NVD by different OCTA-based methods. The tiny buds could not be detected by CFP **A**. en face OCTA images did not show a retinal neovascular network (**B** top), B-scan OCTA showed a small PRHM on the surface of the disc (**B** below). No new blood vessels were indicated by VRI Structure or VRI Angio **C**

**Fig. 3:**
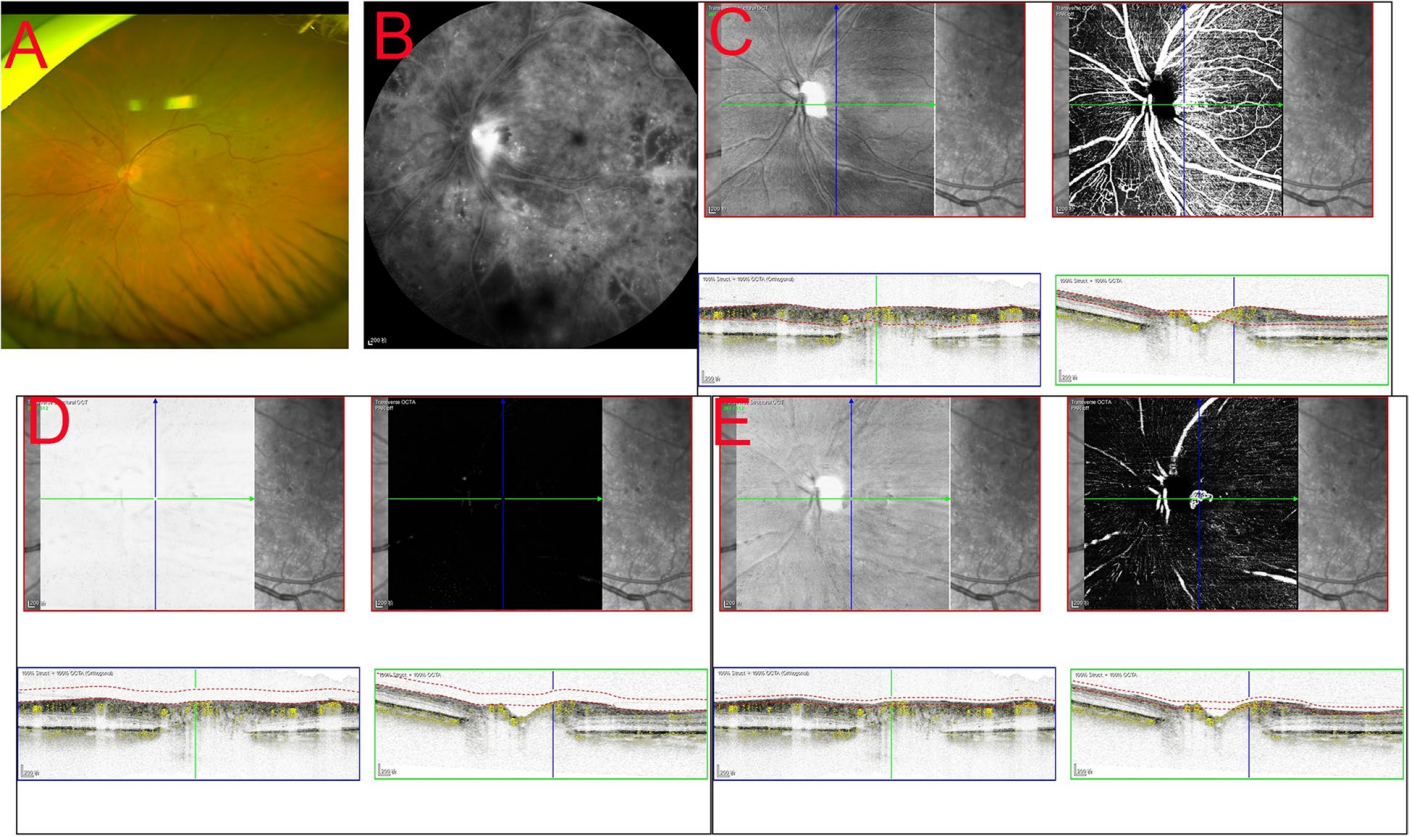
The diagnosis of type III NVD by different OCTA-based methods. It may be difficult to find a new blow vessels clearly by CFP **A**, en face OCTA images and B-scan OCTA **C**, VRI Structure and VRI Angio **D**. However, leakage can be seen on FFA **B**. The structure of new blood vessels can be easily found on the VRI Structure and VRI Angio after manually adjusting the vitreous-retinal interface below the ILM **E**

**Fig. 4:**
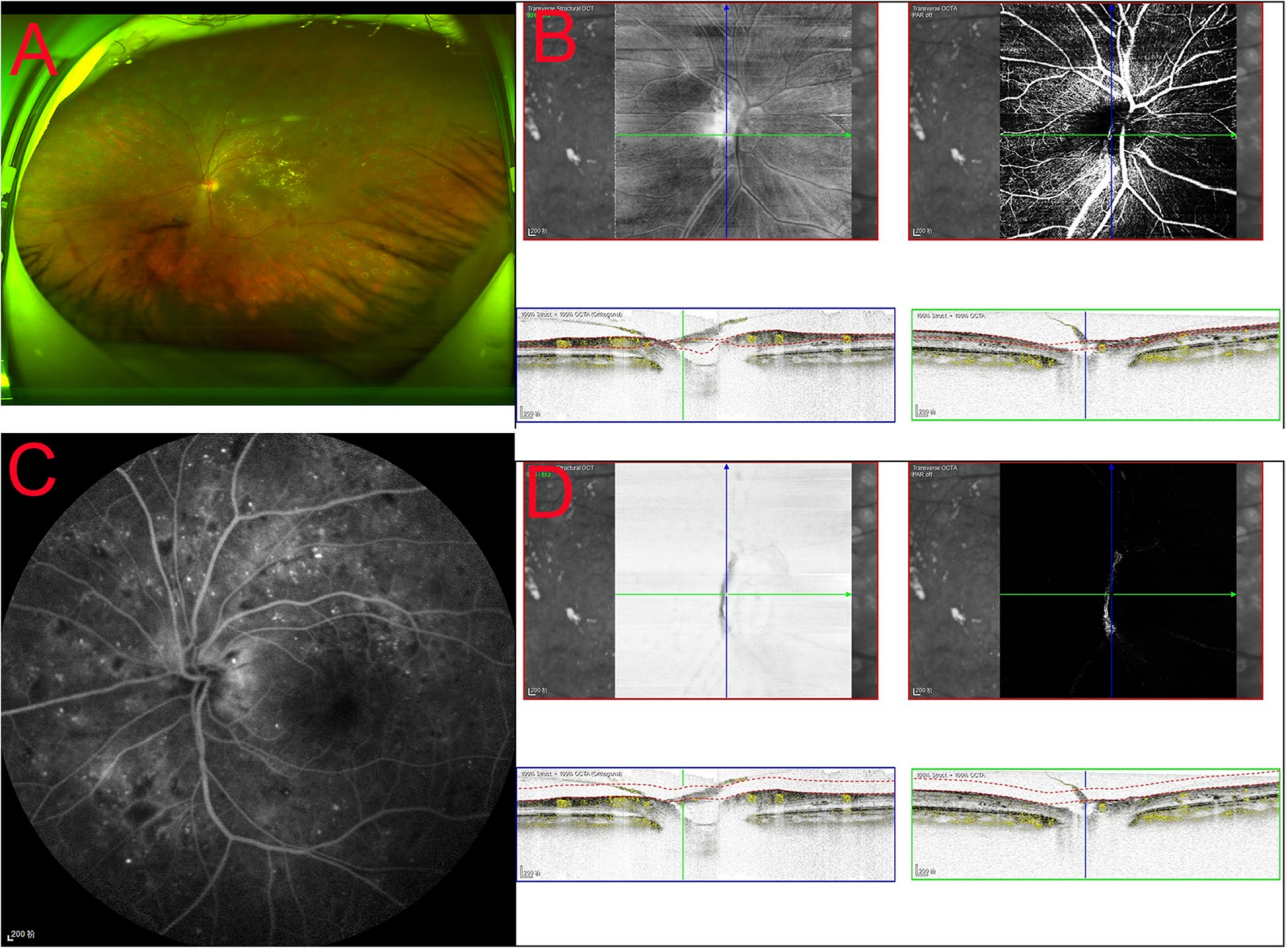
The diagnosis of type IV NVD by different OCTA-based methods. Type IV NVD can be easily detected by CFP **A**, FFA **C**, en face OCTA images and B-scan OCTA **B**, VRI Structure and VRI Angio **D**

Different anatomical subtypes of NVD observed by OCT can explain the decreased detection rate by various methods. Type I NVD encompasses the physiological cup, and it is thus not raised above the disc surface, making it difficult to identify clinically. Similarly, type II NVD, appearing on the B-scan as tiny buds, may be misdiagnosed clinically as dots of haemorrhage. These two types are difficult to detect by en face OCTA (Fig. 2). To our knowledge, the key to the VRI slab method is to identify the vitreous and retinal interface, and the current machine built-in software measurement method makes it more difficult to identify the vitreous retinal interface in the optic disc area (a cup-shaped depression), making it challenging to detect these two types. Similarly, in detecting type III NVD, we found that the boundary of the optic disc in some NVD cases would be difficult to distinguish from the ILM interface. The nonlinear contour of the optic disc or tendency of NVD to invade through the posterior wall may aggravate segmentation errors of the ILM in NVD [27]. By manually separating or adjusting the vitreous-retinal interface below the ILM and then distinguishing the neovascular structure, type III NVD can be detected with a higher accuracy (Fig. 3). However, manual correction may be time-consuming, and a deep learning technique applied to screen and diagnose DR may be significantly needed [28, 29]. Type IV NVD, as this type protrudes towards the vitreous, is easier to identify through fundus photography (Fig. 4).

The present study had a number of shortcomings. First, this was a retrospective study, in which OCTA was compared with clinical examinations. Each patient was examined by an experienced ophthalmologist who confirmed the diagnosis. In addition, although two graders assessed imaging findings, only one senior clinician made the clinical diagnosis for each case. Another shortcoming was that we used the 6*6 scan area. Although the scan range was not wide-angle and was restricted, this study mainly diagnosed NVDs, and the detection of NVE lesions was not performed in other surrounding areas.

Finally, the signals on the VRI slabs were evaluated subjectively, which may have missed more detailed morphological aspects of NV. We also did not distinguish between active and inactive NV. Regardless of activity, however, NV shape and the presence or lack of segmentation faults are more likely to be important for VRI identification.

In conclusion, OCTA and different OCTA-based methods are important for the diagnosis of NVD, and the effectiveness of different detection methods may be related to different types of NVD.

## Data Availability

The datasets used and/or analysed during the current study available from the corresponding author on reasonable request.

## Abbreviations

NVD: Neovascularization in the optic disc
PDR: Proliferative diabetic retinopathy
NV: Neovascularization
IRMAs: Intraretinal microvascular abnormalities
OCTA: Optical coherence tomography angiography
ILM: Inner limiting membrane
VRI: Vitreoretinal interface
CFP: Color fundus photography
FA: Fluorescein angiography
PHPM: Preretinal hyperreflective material (PRHM

## References

[1] Little HL, Zweng HC, Jack RL, Vassiliadis A (1976). Techniques of argon laser photocoagulation of diabetic disk new vessels. Am J Ophthalmol.

[2] Rand  LI,  Prud'homme GJ , Ederer  F, Canner  PL (1985). Factors influencing the development of visual loss in advanced diabetic retinopathy. Diabetic Retinopathy Study (DRS) Report No. 10.. Invest Ophthalmol Vis Sci.

[3] Manjunath V, Papastavrou V, Steel DH, Menon G, Taylor R, Peto T, Talks J (2015). Wide-field imaging and OCT vs clinical evaluation of patients referred from diabetic retinopathy screening. Eye (Lond).

[4] Schwartz R, Khalid H, Sivaprasad S, Nicholson L, Anikina E, Sullivan P, Patel PJ, Balaskas K, Keane PA (2020). Objective Evaluation of Proliferative Diabetic Retinopathy Using OCT. Ophthalmol Retina.

[5] Oishi A, Hidaka J, Yoshimura N (2014). Quantification of the image obtained with a wide-field scanning ophthalmoscope. Invest Ophthalmol Vis Sci.

[6] Wessel MM, Aaker GD, Parlitsis G, Cho M, D'Amico DJ, Kiss S (2012). Ultra-wide-field angiography improves the detection and classification of diabetic retinopathy. Retina.

[7] Silva  PS, Cavallerano  JD, Sun  JK, Noble J, Aiello LM, Aiello LP (2012). Nonmydriatic ultrawide field retinal imaging compared with dilated standard 7-field 35-mm photography and retinal specialist examination for evaluation of diabetic retinopathy. Am J Ophthalmol.

[8] Aiello LP, Odia I, Glassman AR, Melia M, Jampol LM, Bressler NM, Kiss S, Silva PS, Wykoff CC, Sun JK (2019). Comparison of Early Treatment Diabetic Retinopathy Study Standard 7-Field Imaging With Ultrawide-Field Imaging for Determining Severity of Diabetic Retinopathy. JAMA Ophthalmol.

[9] Gass JD (1968). A fluorescein angiographic study of macular dysfunction secondary to retinal vascular disease. IV Diabetic retinal angiopathy Arch Ophthalmol.

[10] Cui Y, Zhu Y, Wang JC, Lu Y, Zeng R, Katz R, Vingopoulos F, Le R, Lains I, Wu DM (2021). Comparison of widefield swept-source optical coherence tomography angiography with ultra-widefield colour fundus photography and fluorescein angiography for detection of lesions in diabetic retinopathy. Br J Ophthalmol.

[11] Parravano M, De Geronimo D, Scarinci F, Querques L, Virgili G, Simonett JM, Varano M, Bandello F, Querques G (2017). Diabetic Microaneurysms Internal Reflectivity on Spectral-Domain Optical Coherence Tomography and Optical Coherence Tomography Angiography Detection. Am J Ophthalmol.

[12] Yasukura S, Murakami T, Suzuma K, Yoshitake T, Nakanishi H, Fujimoto M, Oishi M, Tsujikawa A (2018). Diabetic Nonperfused Areas in Macular and Extramacular Regions on Wide-Field Optical Coherence Tomography Angiography. Invest Ophthalmol Vis Sci.

[13] Arya M, Sorour O, Chaudhri J, Alibhai Y, Waheed NK, Duker JS, Baumal CR (2020). Distinguishing Intraretinal Microvascular Abnormalities from Retinal Neovascularization Using Optical Coherence Tomography Angiography. Retina.

[14] You QS, Guo Y, Wang J, Wei X, Camino A, Zang P, Flaxel CJ, Bailey ST, Huang D, Jia Y (2020). Detection of Clinically Unsuspected Retinal Neovascularization with Wide-Field Optical Coherence Tomography Angiography. Retina.

[15] Wang H, Tao Y (2019). Choroidal structural changes correlate with severity of diabetic retinopathy in diabetes mellitus. BMC Ophthalmol.

[16] Stanga PE, Papayannis A, Tsamis E, Stringa F, Cole T, D'Souza Y, Jalil A (2016). New Findings in Diabetic Maculopathy and Proliferative Disease by Swept-Source Optical Coherence Tomography Angiography. Dev Ophthalmol.

[17] Stanga PE, Tsamis E, Papayannis A, Stringa F, Cole T, Jalil A (2016). Swept-Source Optical Coherence Tomography Angio (Topcon Corp, Japan): Technology Review. Dev Ophthalmol.

[18] Lu ES, Cui Y, Le R, Zhu Y, Wang JC, Lains I, Katz R, Lu Y, Zeng R, Garg I *et al*: Detection of neovascularisation in the vitreoretinal interface slab using widefield swept-source optical coherence tomography angiography in diabetic retinopathy. Br J Ophthalmol 2020. 10.1136/bjophthalmol-2020-317983 PMC9092312 33355148

[19] Khalid H, Schwartz R, Nicholson L, Huemer J, El-Bradey MH, Sim DA, Patel PJ, Balaskas K, Hamilton RD, Keane PA (2021). Widefield optical coherence tomography angiography for early detection and objective evaluation of proliferative diabetic retinopathy. Br J Ophthalmol.

[20] Kong M, Lee MY, Ham DI (2012). Ultrawide-field fluorescein angiography for evaluation of diabetic retinopathy. Korean J Ophthalmol.

[21] Gross JG, Glassman AR, Jampol LM, Inusah S, Aiello LP, Antoszyk AN, Baker CW, Berger BB, Bressler NM, Writing Committee for the Diabetic Retinopathy Clinical Research Network (2015). Panretinal Photocoagulation vs Intravitreous Ranibizumab for Proliferative Diabetic Retinopathy: A Randomized Clinical Trial. JAMA..

[22] Hwang TS, Jia Y, Gao SS, Bailey ST, Lauer AK, Flaxel CJ, Wilson DJ, Huang D (2015). Optical Coherence Tomography Angiography Features of Diabetic Retinopathy. Retina.

[23] Ishibazawa  A, Nagaoka  T, Takahashi A, Omae  T, Tani  T, Sogawa  K, Yokota  H, Yoshida A (2015). Optical Coherence Tomography Angiography in Diabetic Retinopathy: A Prospective Pilot Study. Am J Ophthalmol.

[24] Ishibazawa A, Nagaoka T, Yokota H, Takahashi A, Omae T, Song YS, Takahashi T, Yoshida A (2016). Characteristics of Retinal Neovascularization in Proliferative Diabetic Retinopathy Imaged by Optical Coherence Tomography Angiography. Invest Ophthalmol Vis Sci.

[25] Papayannis A, Tsamis E, Stringa F, Iacono P, Battaglia Parodi M, Stanga PE: Swept-source optical coherence tomography angiography vitreo-retinal segmentation in proliferative diabetic retinopathy. Eur J Ophthalmol 2020:1120672120944028. 10.1177/1120672120944028 32722940

[26] Hirano T, Hoshiyama K, Hirabayashi K, Wakabayashi M, Toriyama Y, Tokimitsu M, Murata T (2020). Vitreoretinal Interface Slab in OCT Angiography for Detecting Diabetic Retinal Neovascularization. Ophthalmol Retina.

[27] Akiyama H, Li D, Shimoda Y, Matsumoto H, Kishi S (2018). Observation of neovascularization of the disc associated with proliferative diabetic retinopathy using OCT angiography. Jpn J Ophthalmol.

[28] GhasemiFalavarjani K, Habibi A, Anvari P, Ghasemizadeh S, Ashraf Khorasani M, Shenazandi H, Sarraf D (2020). Effect of segmentation error correction on optical coherence tomography angiography measurements in healthy subjects and diabetic macular oedema. Br J Ophthalmol.

[29] Ting DSW, Peng L, Varadarajan AV, Keane PA, Burlina PM, Chiang MF, Schmetterer L, Pasquale LR, Bressler NM (2019). Webster DR: Deep learning in ophthalmology: The technical and clinical considerations. Prog Retin Eye Res.

